# Venous thromboembolism is caused by prothrombin p.Arg541Trp mutation in Japanese individuals

**DOI:** 10.1038/s41439-021-00145-x

**Published:** 2021-03-31

**Authors:** Jumpei Yamamoto, Masaya Yamamoto, Kozue Takano, Toru Okazaki, Reiko Arakawa, Hisao Hara, Atsuko Okazaki, Fumihiko Takeuchi, Yukio Hiroi, Norihiro Kato

**Affiliations:** 1 Department of Cardiology, Center Hospital, National Center for Global Health and Medicine, Tokyo, 162-8655 Japan; 2 Department of Genomic Medicine, Center Hospital, National Center for Global Health and Medicine, Tokyo, 162-8655 Japan; 3 Medical Genomics Center, Research Institute, National Center for Global Health and Medicine, Tokyo, 162-8655 Japan; 4 Department of Gene Diagnostics and Therapeutics, Research Institute, National Center for Global Health and Medicine, Tokyo, 162-8655 Japan

**Keywords:** Clinical genetics, Genetics

## Abstract

Venous thromboembolism (VTE) is a multifactorial disease. Because low-frequency variants and rare mutations have been found to predispose carriers toward VTE, there is a need for variant discovery in clinical settings. Therefore, we used a whole-exome approach for a young VTE patient with a positive family history. We identified in the proband and his affected mother a rare, functional missense variant of prothrombin, p.Arg541Trp, which contributes to the clinical picture of VTE.

Venous thromboembolism (VTE) is a potentially deadly condition in which blood clots form in the deep veins of the leg or arm, known as deep-vein thrombosis (DVT), and travel in the circulation to the lungs, known as pulmonary embolism (PE). It is estimated that VTE occurs approximately 1 to 2 per 1000 person-years among people of European descent^1^, with its risk assumed to be lower in Asians^2^. VTE is a multifactorial disease involving genetic and environmental factors and their interaction. The heritability of VTE is estimated to be 35–60%, indicating a substantial genetic basis for the disease^3^. Common genetic variants conferring an increased risk for VTE have been identified by genome-wide association studies^4,5^. Low-frequency variants, e.g., Factor V Leiden (p.R506Q) and F2 (prothrombin) G20210A mutations, and rare mutations in several candidate genes, e.g., *PROC*, *PROS1,* and *SERPINC1* (antithrombin III), have also been found to predispose carriers toward VTE^1^. Of note is the fact that such causal rare mutations have been detected mostly in families showing an autosomal dominant form of inheritance. Accordingly, the need for variant discovery for VTE in clinical settings has been emphasized^6^. In this study, we employed a whole-exome approach for a young patient with a family history of VTE. This study was approved by the institutional ethics review board, and the participants provided written informed consent.

The affected individual of Japanese descent developed acute dyspnea and palpitation during rest at the age of 27 years and was taken to the hospital in an ambulance. When he arrived at the hospital, his oxygen saturation level was 88–90% on room air, and his heart rate was 132 beats/min; there were also signs of right ventricular dysfunction on echocardiography (Fig. 1A), with a markedly elevated plasma D-dimer level (7.5 μg/ml; normal range <1.0 μg/ml). During the last month before the incident, he occasionally felt pain in his left lower leg.

**Fig. 1:**
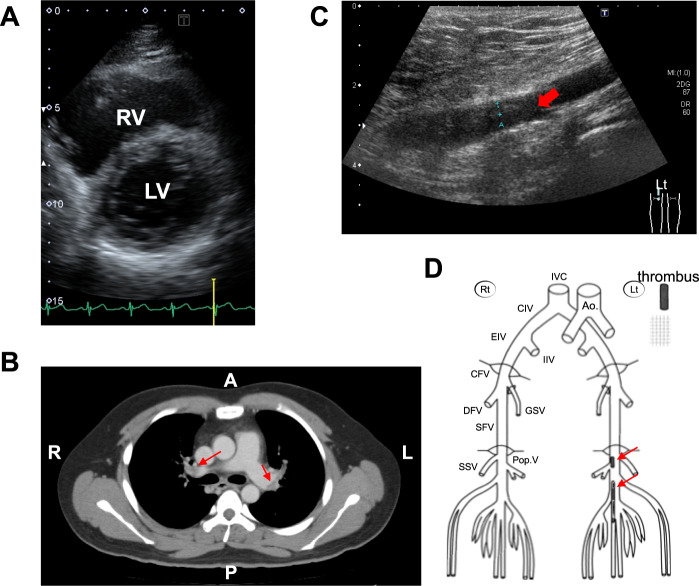
Clinical assessment of the VTE patient. **A** Right ventricular dysfunction found on echocardiography of the proband expressing the prothrombin p.R541W variant. RV, right ventricle; LV, left ventricle. **B** A thoracic CT scan showing multiple pulmonary embolisms (indicated by arrows) from the lobe branch level of the pulmonary artery to the peripheral part of the main pulmonary artery on both sides. **C** Venous ultrasonography of the left leg showing echogenic noncompressable thrombi on the center side of the left popliteal vein. **D** Schematic distribution of deep vein thrombi on the center side of the left popliteal vein and in the portion from the left popliteal vein to the center side of the peroneal vein, as indicated by arrows.

Contrast-enhanced computed tomography (CT) scanning revealed multiple PEs on both sides of the lungs (Fig. 1B). In addition, venous ultrasonography of the legs showed the presence of mural DVT (Fig. 1C, D). The patient was a chronic smoker (20 cigarettes a day since the age of 20 years), was overweight (body mass index, 28.0 kg/m^2^), and mostly did desk work during the day. Furthermore, he was prescribed antidepressants, which were discontinued after hospitalization considering their risk of adverse reactions for thrombosis.

The patient was diagnosed with VTE and underwent anticoagulant therapy with heparin via intravenous administration; after the start of medical treatment, the patient’s conditions promptly improved. Briefly, heparin administration was replaced by oral anticoagulant, and the thromboses steadily reduced thereafter. The patient was discharged on the 13th day of illness. His mother also had recurrent thrombophilia (Fig. 2A), which was initially diagnosed at the age of 27 years during the pregnancy of her second son and recurred at the age of 50 after discontinuance of warfarin; she is currently under prophylactic treatment with an oral anticoagulant. While there appeared to be no other affected blood relatives in the family history, we suspected the involvement of genetic factors in this case.

**Fig. 2:**
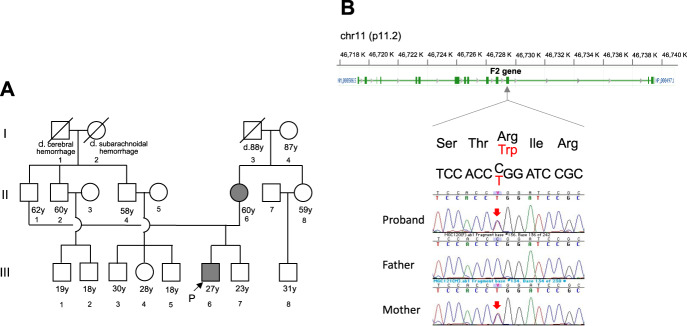
Pedigree and the prothrombin p.R541W variant. **A** Pedigree including the proband (who is indicated with an arrow) and his mother. **B** DNA sequencing chromatogram showing a heterozygous mutation in the F2 gene on human chromosome 11, encoding prothrombin.

Whole-exome sequencing was performed using an Illumina NextSeq 500. Paired-end sequences (2 × 150 bp) were aligned to the human genome reference sequence GRCh37/hg19. Single-nucleotide variations (SNVs) and insertions and deletions were annotated using VarSeq variant analysis software (https://www.goldenhelix.com/products/VarSeq/). We retrieved rare, putatively pathogenic variants, including SNVs that cause nonsynonymous, nonsense, or splice site substitutions, with a particular focus on genes involved in coagulation or fibrinolysis^6^.

Exome sequencing identified a nonsynonymous mutation in the gene encoding prothrombin, p.Arg541Trp or p.R541W (NM_000506.4:c.1621C>T) (Fig. 2B) in the patient (i.e., proband) and his affected mother. Although this mutation has not been registered in ClinVar (https://www.ncbi.nlm.nih.gov/clinvar/) to date, it was recently reported in the Chinese population as a rare functional mutation associated with an increased risk for VTE^7^. In that study, prothrombin p.R541W, exhibiting biased activity toward coagulation, was identified in 3 patients with a family history of VTE from among 374 unrelated VTE patients screened (≈0.8%), whereas it was not detectable in an equal number of healthy subjects of Chinese descent. According to public databases, p.R541W is not found in 8380 Japanese individuals (ToMMo 8.3KJPN, https://jmorp.megabank.tohoku.ac.jp/202008/variants/statistics/) or 141 K individuals of multiethnic origin (gnomAD v2.1.1, https://gnomad.broadinstitute.org). The variant was classified as likely pathogenic according to the ACMG-AMP variant interpretation guidelines, i.e., 1 strong (PS3), 1 moderate (PM2), and 3 supporting (PP1, PP3, and PP4) evidence of pathogenicity^8^.

The F2 gene encodes prothrombin (also known as coagulation factor II), which is proteolytically activated into thrombin by factor Xa. Prothrombin plays a pivotal role in both procoagulant (clot promotion) and anticoagulant (clot inhibition) processes, thereby maintaining hemostatic balance^9^. Genetic impairments of prothrombin can lead to bleeding or thrombosis^10^. Based on functional studies, the prothrombin p.R541W variant results in defective activation of the protein C pathway (i.e., a key anticoagulant system) and is thus responsible for hypercoagulability^7^. In addition to p.R541W, two mutation sites in the F2 (or prothrombin) gene have been established as genetic risk factors for VTE. One site is the residue Arg596-related mutation, initially reported in Japanese patients and showing strong resistance to inhibition by antithrombin^11^; the mutations appear to have a low prevalence (<1% among unrelated VTE patients) in Chinese individuals^12^ and are reported for different ethnic groups^13–15^. Another site is an SNV in the 3’UTR, G20210A, conferring a relatively low risk for VTE;^16^ the frequency of G20210A is ≈2% in Europeans, whereas it is almost absent in Asians^17^.

Our identification of the prothrombin p.R541W variant in VTE patients of Japanese descent is noteworthy at least regarding the following three points. First, the functional p.R541W variant is reproducibly detected in Chinese and Japanese, indicating that it is not restricted to a particular region (or country) but is shared among East Asians; however, it should be further investigated whether the mutation is rare in other ethnic groups. Second, heterozygous p.R541W carriers are considered to need medical follow-up, including prophylactic anticoagulation, during a certain period of time, as there is a risk for recurrent VTE, as observed for the patient’s mother in this study. Medical follow-up should be performed with more caution when mutation carriers are exposed to additional, acquired risk factors for VTE (e.g., antidepressants and pregnancy in the present case). Third, variant discovery analysis by targeted gene panel testing or whole-exome sequencing helps to clarify genetic factors, in particular, causal rare mutations, in VTE patients with a family history.

## Data Availability

The relevant data from this Data Report are hosted at the Human Genome Variation Database at 10.6084/m9.figshare.hgv.2981

## Data availability

The deidentified participant data will not be shared except those presented in the manuscript.

## References

[1] Crous-Bou M, Harrington LB, Kabrhel C (2016). Environmental and genetic risk factors associated with venous thromboembolism. Semin Thromb. Hemost..

[2] Zakai NA, McClure LA (2011). Racial differences in venous thromboembolism. J. Thromb. Haemost..

[3] Tregouet DA, Morange PE (2018). What is currently known about the genetics of venous thromboembolism at the dawn of next generation sequencing technologies. Br. J. Haematol..

[4] Klarin D (2019). Genome-wide association analysis of venous thromboembolism identifies new risk loci and genetic overlap with arterial vascular disease. Nat. Genet..

[5] Desch KC (2020). Whole-exome sequencing identifies rare variants in STAB2 associated with venous thromboembolic disease. Blood.

[6] Lee EJ (2017). Whole-exome sequencing in evaluation of patients with venous thromboembolism. Blood Adv..

[7] Wu X (2020). Prothrombin Arg541Trp mutation leads to defective PC (Protein C) pathway activation and constitutes a novel genetic risk factor for venous thrombosis. Arterioscler Thromb. Vasc. Biol..

[8] Richards S (2015). Standards and guidelines for the interpretation of sequence variants: a joint consensus recommendation of the American College of Medical Genetics and Genomics and the Association for Molecular Pathology. Genet. Med..

[9] Crawley JT, Zanardelli S, Chion CK, Lane DA (2007). The central role of thrombin in hemostasis. J. Thromb. Haemost..

[10] Ding Q, Yang L, Zhao X, Wu W, Wang X, Rezaie AR (2017). Paradoxical bleeding and thrombotic episodes of dysprothrombinaemia due to a homozygous Arg382His mutation. Thromb. Haemost..

[11] Miyawaki Y (2012). Thrombosis from a prothrombin mutation conveying antithrombin resistance. N. Engl. J. Med..

[12] Wu X (2018). Screening and functional exploration of prothrombin Arg596 related mutations in Chinese venous thromboembolism patients. J. Clin. Pathol..

[13] Bulato C (2016). New prothrombin mutation (Arg596Trp, Prothrombin Padua 2) associated with venous thromboembolism. Arterioscler. Thromb. Vasc. Biol..

[14] Miljic P (2017). Clinical and biochemical characterization of the prothrombin Belgrade mutation in a large Serbian pedigree: new insights into the antithrombin resistance mechanism. J. Thromb. Haemost..

[15] Kishimoto M (2016). The first case of antithrombin-resistant prothrombin Belgrade mutation in Japanese. Ann. Hematol..

[16] Poort SR, Rosendaal FR, Reitsma PH, Bertina RM (1996). A common genetic variation in the 3’-untranslated region of the prothrombin gene is associated with elevated plasma prothrombin levels and an increase in venous thrombosis. Blood.

[17] Huang SS, Liu Y, Jing ZC, Wang XJ, Mao YM (2016). Common genetic risk factors of venous thromboembolism in Western and Asian populations. Genet. Mol. Res..

